# A systematic bias in float pH leads to overestimation of derived *p*CO_2_ and underestimation of carbon uptake by the Southern Ocean

**DOI:** 10.1038/s41598-026-43863-4

**Published:** 2026-03-17

**Authors:** Chuqing Zhang, Yingxu Wu, Peter J. Brown, David Stappard, Amavi N. Silva, Toby Tyrrell

**Affiliations:** 1 School of Ocean and Earth Science, University of Southampton, National Oceanography Centre Southampton, Southampton, UK; 2 Polar and Marine Research Institute, Jimei University, Xiamen, China; 3 National Oceanography Centre, Southampton, UK; 4 GEOMAR Helmholtz Centre for Ocean Research Kiel, Kiel, Germany

**Keywords:** Biogeochemistry, Environmental sciences, Ocean sciences

## Abstract

The carbon flux estimated from biogeochemical Argo float data indicates a lower annual carbon uptake by the Southern Ocean compared to fluxes derived from other observations (e.g., ship and aircraft measurements). The root cause of this discrepancy remains controversial, with growing evidence suggesting that potential biases in float-derived *p*CO_2_ may be a plausible explanation. Here, we perform a multi-variable comparison of vertical profiles between float- and ship based-data and reveal consistent discrepancies in pH, *p*CO_2_ and dissolved inorganic carbon, which are not found in other variables such as dissolved oxygen, nitrate and total alkalinity. Our findings are consistent with a previously unrecognized negative bias in float pH driving a positive offset in float-derived *p*CO_2_. The float-derived surface *p*CO_2_ is, on average, biased high by 15 ± 3 µatm compared to ship data, representing a larger magnitude of bias than previously recognized. Biases exist in both surface and deep waters, including old deep waters containing minimal anthropogenic carbon. A more sophisticated adjustment for float pH, involving multiple cross-reference depths, may be required for accurate estimation of air-sea CO_2_ exchange in the Southern Ocean.

**Supplementary Information:**

The online version contains supplementary material available at 10.1038/s41598-026-43863-4.

## Introduction

A biogeochemical (BGC) Argo float is an advanced autonomous monitoring platform, equipped with additional sensors (extra to those on standard Argo floats) to measure biogeochemical variables^1–3^ including pH, dissolved oxygen (O_2_), and nitrate (NO_3_). Under normal operating conditions it will collect data on vertical profiles (0–2000 m), carried out once every 5–10 days^4^. Floats have garnered significant interest for their autonomous sampling capabilities, high-frequency of sampling, and ability to observe remote regions. The Southern Ocean (SO) has historically been one of the least observed ocean basins because its environment is harsh, especially in winter, presenting difficulties for ship-based sampling^5^. However, it plays a critical role in the global heat and carbon cycles as well as the Earth’s climate^6^; it accounts for ~ 40% of heat uptake and ~ 44% of anthropogenic carbon uptake by the global ocean^7^. To compensate for this, the Southern Ocean Carbon and Climate Observations and Modeling (SOCCOM) project had deployed over 260 BGC Argo floats in the SO by the end of 2023 to enhance observational capability^8,9^.

Accurate calculation of air–sea carbon dioxide (CO_2_) fluxes has profound implications for understanding the carbon cycle and the ocean carbon sink^10^. The CO₂ flux is typically estimated as the product of the gas transfer velocity, the solubility of CO₂ in seawater, and the difference in partial pressure of CO₂ (*p*CO_2_) between the seawater and atmosphere. Previous calculations of CO_2_ fluxes were based on high-quality ship data and model estimates^11–13^. The sparse ship observations in the SO result in larger uncertainties in air-sea CO_2_ fluxes than in other ocean basins^14,15^. The BGC Argo floats deployed by SOCCOM have generated a substantial volume of data with broad spatial and temporal coverage, with great potential for improved estimation of the SO CO_2_ flux. Float-based *p*CO_2_ in seawater is calculated from a combination of measured pH and algorithm-estimated total alkalinity (TA)^16–19^, therefore introducing multiple sources of uncertainty^16^.

Float-based CO_2_ annual fluxes indicate a considerably lower carbon uptake by the SO than those obtained through other approaches (such as from ship-based measurements or model estimations), thereby challenging conventional views^18,20^. This discrepancy has been attributed to a scarcity of ship data during winter—when high-latitude *p*CO_2_ values peak due to intensive upwelling and deep water entrainment^18,20^. However, two subsequent independent studies have questioned this explanation: Winter outgassing estimated by extrapolation from summertime ship observations is markedly lower than the fluxes derived from float observations^21^. Moreover, estimation using data collected from aircraft of the CO_2_ flux south of 45°S suggest an annual ingassing to the ocean of 0.53 ± 0.23 Pg C yr^− 1 22^. This aircraft-derived estimate aligns closely with ship-based results^22–24^, whereas the float-based estimate is an annual outgassing of 0.3 Pg C yr^− 1^. Consequently, the float and aircraft data yield contradictory conclusions of the total annual air–sea CO₂ flux in the SO, with float data indicating net outgassing and aircraft data suggesting net ingassing^20,22,25^.

One potential explanation for the discrepancy in estimating SO carbon fluxes is that the float pH and derived *p*CO_2_ data may be systematically offset from their true values. Over the past few years, the possibility of such a bias has become a topic of ongoing debate. For instance, a crossover analysis with the Surface Ocean CO₂ Atlas (SOCAT) data reported an average positive bias of 3.6 µatm in surface float *p*CO_2_^18^. Similarly, a meta-analysis of CO_2_ and O_2_ concentrations identified offsets that resulted in float-derived *p*CO_2_ values being biased high by ~ 5.8 µatm at the surface^26^. In the Drake Passage, a mean surface *p*CO_2_ discrepancy of 14 µatm has been observed, with float *p*CO_2_ being consistently higher than ship observations, a magnitude that cannot be fully attributed to upwelling effects^27^. Moreover, long-term mooring data from the subantarctic region found that float *p*CO_2_ was 3–7 µatm higher than observed values^28^. Critically, the current float pH adjustment method has also been questioned, with suggestions that the correction should be pH-dependent rather than a fixed offset^29^. Incorporating a second reference point closer to the surface in the float pH correction process has been proposed to improve the accuracy of float pH measurements^30^. Other studies have similarly reported varying degrees of bias in float-derived *p*CO_2_: float-to-reference *p*CO_2_ offsets were reported to span from − 4.6 ± 4.1 µatm to + 8.6 ± 4.0 µatm in subtropical cruises^31^; +2.6 ± 12.8 µatm during a South Georgia bloom^32^; from + 4 to + 9.5 µatm in Subantarctic Mode Water formation regions^33^; more than + 11 µatm compared with a machine learning reconstruction^34^(positive values indicate that the float‑derived *p*CO_2_ is higher than the reference, while negative values indicate it is lower).

Previous investigations of the accuracy of float-derived *p*CO_2_ have been hampered by several limitations: the comparisons (i) primarily focused on localized regions, raising concerns about their applicability to the entire SO, (ii) solely concentrated on surface data, thereby neglecting potential biases in subsurface *p*CO_2_ and, (iii) were restricted to instances when a ship and a float are co-located in time and space—typically immediately after float deployment—resulting in the use of less than 1% of the available data. Additionally, (iv) the evaluations have only considered *p*CO_2_ without concurrently assessing the accuracy of other related variables. To address these shortcomings and provide a comprehensive evaluation of float pH and derived *p*CO_2_, we carried out a basin-scale, quantitative, multi-variable, whole-profile comparison between float and ship data across the SO (south of 35°S), using the Global Ocean Data Analysis Project (GLODAP) as reference. GLODAP provides high-quality, internally consistent measurements and is widely used as a benchmark for carbonate system data. While numerous efforts have examined float carbonate system data quality, no consensus has been reached regarding the cause of observed discrepancies. This study seeks to address several unresolved questions: (i) Do float carbonate system data exhibit significant systematic biases? (ii) Are these biases limited to the surface ocean or do they extend to the subsurface? (iii) If present, what is the magnitude of these biases? (iv) What recommendations are proposed for future corrections of float carbonate system data? The results presented here provide new insights into these questions.

## Methods

The Southern Ocean is defined as south of 35°S in this study.

### Dataset descriptions

#### Float data

The BGC Argo float data were downloaded from https://library.ucsd.edu/dc/object/bb51655605. This snapshot contains all data before 2023-12-20 from SOCCOM floats, GO-BGC (Global Ocean Biogeochemistry Array) floats and also from additional University of Washington/MBARI floats^35^. The data were subjected to the delayed-mode quality control (DMQC) at MBARI following Maurer et al.^36^. The float variables that are relevant to this study are: pH (in-situ temperature and pressure, on the total scale)/(25 °C, 0 dbar, on the total scale), *p*CO_2_, dissolved inorganic carbon (DIC), O_2_, NO_3_, in situ temperature (T), practical salinity (S) and TA. Only the data for which quality flags are good (0) for all of these variables were used. 10,003 profiles from 176 floats in the SO were chosen for final analysis. In the following, all pH data are reported on the total scale; temperature and pressure conditions (in-situ or 25 °C, 0 dbar) are used to distinguish them.

The GO-BGC adjustment procedure for float pH data is based on crossover analysis in deep waters between float and shipboard measurements^37^. Firstly, an empirical algorithm is developed for estimating pH_in−situ_ (as a function of T, S, P, and O_2_) from shipboard bottle measurements at depths of 1000–2000 m. Then this algorithm is applied to float T, S, P and O_2_ in order to generate an expected float pH_in−situ_ value at 1500 m depth. Finally, the measured float pH_in−situ_ and the expected float pH_in−situ_ are compared at 1500 m depth and the resulting pH_in−situ_ offset is applied to adjust the entire float profile. This correction approach assumes that the primary float pH_in−situ_ bias arises from a pressure-independent offset in the sensor’s reference potential, resulting in a consistent error across depths, as supported by previous studies on sensor behavior and deep-ocean stability^16,36,38^.

An additional adjustment^16^ is applied when calculating *p*CO_2_ from float pH (Eq. 1) in following order: (1) float measured pH_in−situ_ is converted to pH_25 °C, 0 dbar_ using CO2SYS with LIAR-estimated TA and in-situ temperature, salinity and pressure, following the standard SOCCOM processing^35^. (2) Using Eq. (1) and the 1500 m depth pH_25 °C, 0 dbar_, a correction is calculated for each profile. (3) This is added to each pH value in the profile before finally calculating *p*CO_2_. This empirical, pH-dependent correction which is applied for the calculation of *p*CO_2_ (but not for other carbon variables), is intended to compensate for the systematic bias in *p*CO_2_ calculated from float pH and LIAR-estimated TA relative to *p*CO_2_ derived from shipboard DIC and TA (which is consistent with direct *p*CO_2_ measurements), a bias that arises from internal inconsistencies in inorganic carbon thermodynamics^16^.

1 $$pHadjustment=-0.034529\times{pH}_{25^\circ\:C,0dbar}+0.26709$$

The float *p*CO_2_ was converted from measured-adjusted pH and locally interpolated alkalinity regression (LIAR) algorithm-estimated TA. The LIAR algorithm has a reported uncertainty^19^ of 5.6 µmol kg^−1^. The conversion from pH_in−situ_ and TA to float *p*CO_2_ was calculated using the Matlab version for CO2SYSv3^39^, based on the following assumptions: (1) pH is on the total scale. (2) K_1_ and K_2_ dissociation constants were taken from Lueker et al.^40^. (3) The KSO_4_ dissociation constant was taken from Dickson^41^. (4) The borate to salinity ratio was taken from Lee et al.^42^. (5) The KF dissociation constant was taken from Perez and Fraga^43^. Silicate and phosphate concentrations were not measured by the floats; however, estimates based on Redfield ratios^44^ improved the carbonate system calculations. If a nitrate value was considered to be of good quality, then silicate = nitrate×2.5 and phosphate = nitrate/16; otherwise the best estimate for both was considered to be 0, following standard SOCCOM processing^35^.

Williams et al. identified a large number of potential sources of float *p*CO_2_ uncertainty, which they grouped into 3 categories^16^: (1) uncertainties in pH sensors, (2) uncertainties in derived alkalinity estimates, and (3) uncertainties in carbonate system equilibrium constants, with category (1) thought to have the greatest contribution. The uncertainty of each individual float *p*CO_2_ value was estimated to be 2.7% (11 µatm at *p*CO_2_ of 400 µatm)^16^.

### Ship data (GLODAP)

Ship data (GLODAP) were obtained from https://www.glodap.info (last access: 05 February 2024). The GLODAPv2.2023 dataset contains almost 1.4 million internally-consistent samples of biogeochemical variables, globally collected on 1,108 cruises during the period 1972–2023^45^. The ship data variables that are relevant to this study are: pH_in−situ_, pH_25 °C, 0 dbar_, DIC, O_2_, NO_3_, T, S, silicate, phosphate, and TA. Only the sampling points where all the secondary quality flags for these variables are good (flag value of 1) were selected. 2,862 profiles in the SO containing 42,691 sampling points were included in the final dataset.

Ship *p*CO_2_ was calculated from GLODAP’s DIC and TA data, typically directly measured from samples taken from Niskin bottles^46^. The procedure for converting ship DIC and TA to ship *p*CO_2_ also employed the Matlab version of CO2SYSv3^39^, with the same assumptions as above (i.e., for float *p*CO_2_)^40–43^. Silicate and phosphate were taken from direct measurements. The uncertainty in each individual ship *p*CO_2_ value is reported to be ~ 12 µatm at *p*CO_2_ of 400 µatm^34^.

### Gridded data (OceanSODA-ETHZ)

OceanSODA-ETHZ (referred as ‘OceanSODA’ in this manuscript) is a global gridded data set of the surface ocean carbonate system. This dataset is at a monthly resolution over the period 1985 through 2022 at a spatial resolution of 1°×1°, containing multiple variables in the surface ocean, i.e., DIC, TA, *p*CO_2_, pH_in−situ_, and the saturation state with respect to calcium carbonate (CaCO_3_). Its *p*CO_2_ is based mainly on data from the Surface Ocean CO_2_ Atlas (SOCAT) data^23,47,48^, with uncertainty of 14 µatm^49^.

### Matched float-ship data

This research focuses on comparing data along the entire vertical profile (0–2000 m, the sampling depth range of floats); subsurface water masses should exhibit little or no seasonality and minimal interannual variation, therefore subsurface matches between float and ship data were established based on depth and distance, disregarding time. The distance match window was 25 km; if the float profile was within 25 km of the ship profile, the two profiles were matched. If multiple ship CTD (Conductivity‑Temperature‑Depth) casts were matched to a single float profile, only the spatially closest cast was selected. The depth-based matching was carried out as follows: A 10 m depth match window was used for depths shallower than 200 m, while a 100 m window was used for depths deeper than 200 m, to account for larger sampling intervals in the subsurface. If there were multiple matched points, only the point with the smallest depth difference was selected. Finally, we obtained 724 matched profiles containing a total of 11,183 matched float-ship data points (Supplementary Fig. 1). Each point contains the corresponding float and ship variables: pH_in−situ_, pH_25 °C, 0 dbar_, *p*CO_2_, O_2_, NO_3_, T, S and TA. The average depth difference of data points at depths shallower than 200 m was 1.8 m; for those at depths deeper than 200 m, it was 13.5 m. Given the small depth differences, it is reasonable to expect float and ship data to be similar in value, if measured accurately, especially in the subsurface.

## Methods for comparing matched float-ship data in subsurface water

### Anthropogenic carbon calculation

Float data have generally been collected more recently than ship data, and thus, where in contact with the atmosphere, are expected to contain more anthropogenic carbon. This leads to a potentially confounding effect on our comparisons, mistaking different amounts of anthropogenic carbon for measurement bias. In this study, we therefore paid particular attention to water masses with minimal anthropogenic carbon perturbation (unacidified waters), which serve as a baseline for assessing float carbonate system variables’ data quality, independent of long-term ocean acidification trends. These water masses are primarily Upper Circumpolar Deep Waters and Lower Circumpolar Deep Waters (UCDW and LCDW, respectively); the sources of the UCDW are Indian and Pacific Deep Waters (IDW and PDW, respectively) while the LCDW is primarily fed by North Atlantic Deep Water (NADW). These water masses have been isolated from the atmosphere since preindustrial times and thus are minimally influenced by anthropogenic carbon^50–52^. This focus on old, deep waters is a key analytical strategy which ensures that any discrepancies observed between float and ship-based data are not attributable to changes in ocean chemistry driven by anthropogenic carbon. Therefore, we used estimates of anthropogenic carbon as one indicator to isolate matched float-ship data that are unaffected by ocean acidification. To establish the accuracy of the calculated anthropogenic carbon in the matched float-ship data, we employed both ∆C* and TrOCA approaches for estimation.

### ∆C^*^ approach

∆C* has been used extensively to calculate anthropogenic carbon^53–56^. The specific calculation process is as follows:

2 $$\varDelta{C}^{*}={C}_{m}-{C}_{280}-\varDelta{C}_{bio}$$

3 $${C}_{anth}={C}_{m}-{C}_{280}-\varDelta{C}_{bio}-\varDelta{C}_{dis}=\varDelta{C}^{*}-\varDelta{C}_{dis}$$

4 $${C}_{280}=CO2SYS(S,T,{TA}^{0},280)$$

5 $${TA}^{0}=378.1+55.22*S+0.0716*PO-1.236*\theta$$

6 $$\varDelta{C}_{bio}=-117/170({O}_{2}-{O}_{2,sat})+1/2(TA-{TA}^{0}-16/170({O}_{2}-{O}_{2,sat}\left)\right)-106/104{N}_{anom}^{*}$$

where C_anth_ denotes the anthropogenic carbon concentration; C_m_ denotes measured DIC; C_280_ denotes the DIC of waters in equilibrium with an atmospheric CO_2_ concentration of 280 µatm; ∆C_bio_ denotes the DIC changes due to remineralization of organic matter and dissolution of calcium carbonate particles in the time since the parcel of water left the surface; TA^0^ donates the preformed TA, based on a multiple linear regression fit of TA values in the Antarctic region^57^; ∆C_dis_is the air-sea disequilibrium component taken from Sabine et al.^58^. PO is a quasi-conservative tracer^59^. N^*^_anom_ is the N^*^ anomaly from the mean, *θ* is the potential temperature, and O_2,sat_ is the saturated O_2_ concentration.

### TrOCA approach

The TrOCA (Tracer combining O_2_, DIC, TA) method is also a widely adopted approach for estimating anthropogenic carbon^60–62^. The specific calculation process is shown in the following equations:

7 $$TrOCA={O}_{2}+1.279(DIC-1/2TA)$$

8 $${TrOCA}^{0}={e}^{(7.511-\left(1.087\times{10}^{-2}\right)\theta-7.81\times{10}^{5}/{TA}^{2})}$$

9 $${C}_{anth}=\left(TrOCA-{TrOCA}^{0}\right)/1.279$$

where C_anth_ denotes the anthropogenic carbon concentration, *θ* is potential temperature, TrOCA is the conservative tracer as originally defined by Touratier and Goyet^60,62^, and TrOCA^0^ represents the pre-industrial TrOCA value, derived by fitting a combined dataset of ∆¹⁴C- and CFC-11-based estimates to an exponential function (Eq. 8), using the GLODAP world ocean database as a reference^56^.

Ultimately, the anthropogenic carbon values calculated using both methods show strong agreement (Supplementary Fig. 2) and can be used reliably to identify water masses that are minimally influenced by anthropogenic carbon.

### Identification of unacidified subsurface water for matched float-ship data

We applied a three-fold constraint to carefully identify ‘old’ subsurface waters (UCDW and LCDW) that are largely unaffected by ocean acidification (minimal anthropogenic carbon signal): (1) depth > 200 m. (2) temperature and potential density values characteristic of either UCDW (2.5℃< temperature < 3℃, 27.4 < potential density < 27.7) or LCDW (0℃< temperature < 2.5℃, potential density > 27.7). (3) ship-based C_ant_ < 10 µmol kg^− 1^ (For T-S diagrams of ship data and float data, see Supplementary Fig. 3). We did not use float-derived estimates of anthropogenic carbon for screening, as any biases in float-based pH, *p*CO_2_, and DIC data (as we conclude in this paper) compromise the reliability of their values.

## Methods for identifying float *p*CO_2_ bias in surface water

### CORS plot (carbon and oxygen relative to saturation)

The CORS method is a CO_2_-O_2_ analysis technique which involves comparing the deviations of O_2_ and CO_2_concentrations from their respective saturation levels. Following Wu, et al.^26^, we adopted an identical approach for both O_2_ and CO_2_ and, proceeded to compare the observed dissolved concentrations of O_2_ and CO_2_ ($\left[{O}_{2,observed}\right]$ and $\left[{CO}_{2,observed}\right]$ respectively) in surface seawater with their respective saturation values ($\left[{O}_{2,saturation}\right]$ and $\left[{CO}_{2,saturation}\right]$). The saturation values represent the points at which the net air-sea gas exchange rate should be zero for each gas. Differences from saturation of O_2_ and CO_2_ are calculated following:

10 $$\varDelta{O}_{2}=\left[{O}_{2,observed}\right]-\left[{O}_{2,saturation}\right]$$

11 $$\varDelta{CO}_{2}=\left[{CO}_{2,observed}\right]-\left[{CO}_{2,saturation}\right]$$

The saturation concentration of O_2_ was calculated using Garcia & Gordon’s Eqs.^63,64^; however, while they calculated the O_2_ saturation concentration at an assumed 1 atm of atmospheric pressure (Eq. 12), we instead used the local in-situ sea level pressure (SLP, usually < 1 atm in the SO) as given in Eq. (13):

12 $$\left[{O}_{2,saturation}^{1atm}\right]={K}_{{O}_{2}}\times\:p{O}_{2}^{1atm}={K}_{{O}_{2}}\times\:x{O}_{2,air}\times\left({P}_{1atm}-{P}_{Sw}\right)$$

13 $$\left[{O}_{2,saturation}^{SLP}\right]={K}_{{O}_{2}}\times\:p{O}_{2}^{SLP}={K}_{{O}_{2}}\times\:x{O}_{2,air}\times\left({P}_{SLP}-{P}_{Sw}\right)$$

Here, the subscripts ‘1 atm’ and ‘SLP’ indicate two different pressures. ${K}_{{O}_{2}}$ is the solubility of dissolved O_2_, ${O}_{2,saturation}^{1atm}$ is the result based on the Garcia & Gordon’s Eqs.^63,64^. ${O}_{2,saturation}^{SLP}$ is the result based on our modified equation. ${P}_{Sw}$ is the water vapor pressure calculated from the surface temperature and salinity^65^. ${P}_{SLP}$ was taken from the monthly gridded climate data downloaded from National Oceanic and Atmospheric Administration (NOAA) (Gridded Climate: NOAA Physical Sciences Laboratory, last access: 10 July 2023). Combining Eqs. (12) and (13):

14 $$\left[{O}_{2,saturation}^{SLP}\right]=\left[{O}_{2,saturation}^{1atm}\right]\times\left({P}_{SLP}-{P}_{Sw}\right)/\left({P}_{1atm}-{P}_{Sw}\right)$$

$\left[{CO}_{2,saturation}\right]$ was calculated with Henry’s equation. ($\left[{CO}_{2,observed}\right]={K}_{{CO}_{2}}\times{pCO}_{2,equilibrium})$, where ${pCO}_{2,equilibrium}$ is the partial pressure of CO_2_ in seawater at equilibrium with atmospheric CO_2_ (Eq. 15).

15 $${pCO}_{2,equilibrium}=x{CO}_{2,air}\times\left({P}_{SLP}-{P}_{Sw}\right)$$

where $x{CO}_{2,air}$ is the mole fraction of CO_2_ (ppm) in dry atmosphere. The monthly mean atmospheric $x{CO}_{2,air}$ values from the monitoring site at Palmer Station, Antarctica were used for the SO (downloaded from https://www.esrl.noaa.gov/gmd/ccgg/trends/, last access: 14 July 2023). The solubility of CO_2_ $\left({K}_{{CO}_{2}}\right)$ was calculated from the formula proposed by Weiss^66^.

### Matched float-OceanSODA data

Since the surface ocean is defined as shallower than 5 m by both SOCAT and OceanSODA, we selected all float surface data in the top 5 m for which the quality flag is good for *p*CO_2_. Because of the strong spatial and temporal variability in surface waters, float data and OceanSODA were only matched when from the same month, same year and within 25 km of each other. The final set of matched float-OceanSODA data contains surface data from 594 float profiles and matching OceanSODA data.

## Methods for statistical analysis of data

### Deming regression

Deming regression^67^ is a technique used to fit a straight line to two-dimensional data where both variables, X and Y, are measured with error. This method contrasts with simple linear regression, where only the response variable, Y, is considered to be subject to measurement error. Applying this method to compare the matched float-ship data takes account of errors in observations in both ship and float data.

### Cohen’s d

Cohen’s d^68^ is a standardized measure of effect size used to quantify the difference between two group means, which scales the difference in the two means to the inherent variability of the data. Specifically in this work, we applied Cohen’s d to evaluate the deviation degree of different matched variables. It is calculated as:

16 $$DD=\frac{{M}_{F}-{M}_{S}}{\sqrt{{{SD}_{F}}^{2}+{{SD}_{S}}^{2}}}$$

where M_F_ is the mean value of float data, M_S_ is the mean value of ship data, SD_F_ is the standard deviation of float data and SD_S_ is the standard deviation of ship data. A negative value of DD indicates that the float mean is smaller than the ship mean and vice versa. The absolute value of DD represents the magnitude of this difference, normalized by the variability of the data.

### Monte Carlo simulation process

We applied Monte Carlo simulation^69^ to calculate the impact of uncertainties in individual float and ship data on the mean differences of matched float-ship *p*CO_2_. The uncertainty of an individual float *p*CO_2_ value is 11 µatm^16^ when float *p*CO_2_ is 400 µatm. The uncertainty of ship *p*CO_2_ is 12 µatm^34^ at 400 µatm. The probability density function of average float *p*CO_2_ and ship *p*CO_2_ from 1000 Monte Carlo iterations as well as the difference between the two was calculated for an example case. The following procedure was used: (1) the average float *p*CO_2_ was assumed to be 400 µatm while that of the ship *p*CO_2_ was assumed to be 383 µatm (according to the mean Δ*p*CO_2_ in 200–1500 m depth). (2) Where ‘G(µ,σ)’ is a random number from a normal (Gaussian) distribution with mean of µ and standard deviation of σ, 724 independent float *p*CO_2_ values, each equal to 400 + G(0,11) were generated. Similarly, 724 independent ship *p*CO_2_ values, each equal to 383 + G(0,12), were also generated. The sample size ‘724’ in both cases is equal to the amount of ship and float profiles used in the study. (3) For each pair of float and ship values, individual *p*CO_2_ difference was calculated. This was then followed by the computation of the average of the 724 differences, (4) The steps (1) to (3) were repeated 1000 times to obtain 1000 average differences, (5) the corresponding frequency distribution plot of the average differences was constructed.

## Results

### Consistencies of variables between matched float-ship data in subsurface waters with minimal anthropogenic carbon

**Fig. 1:**
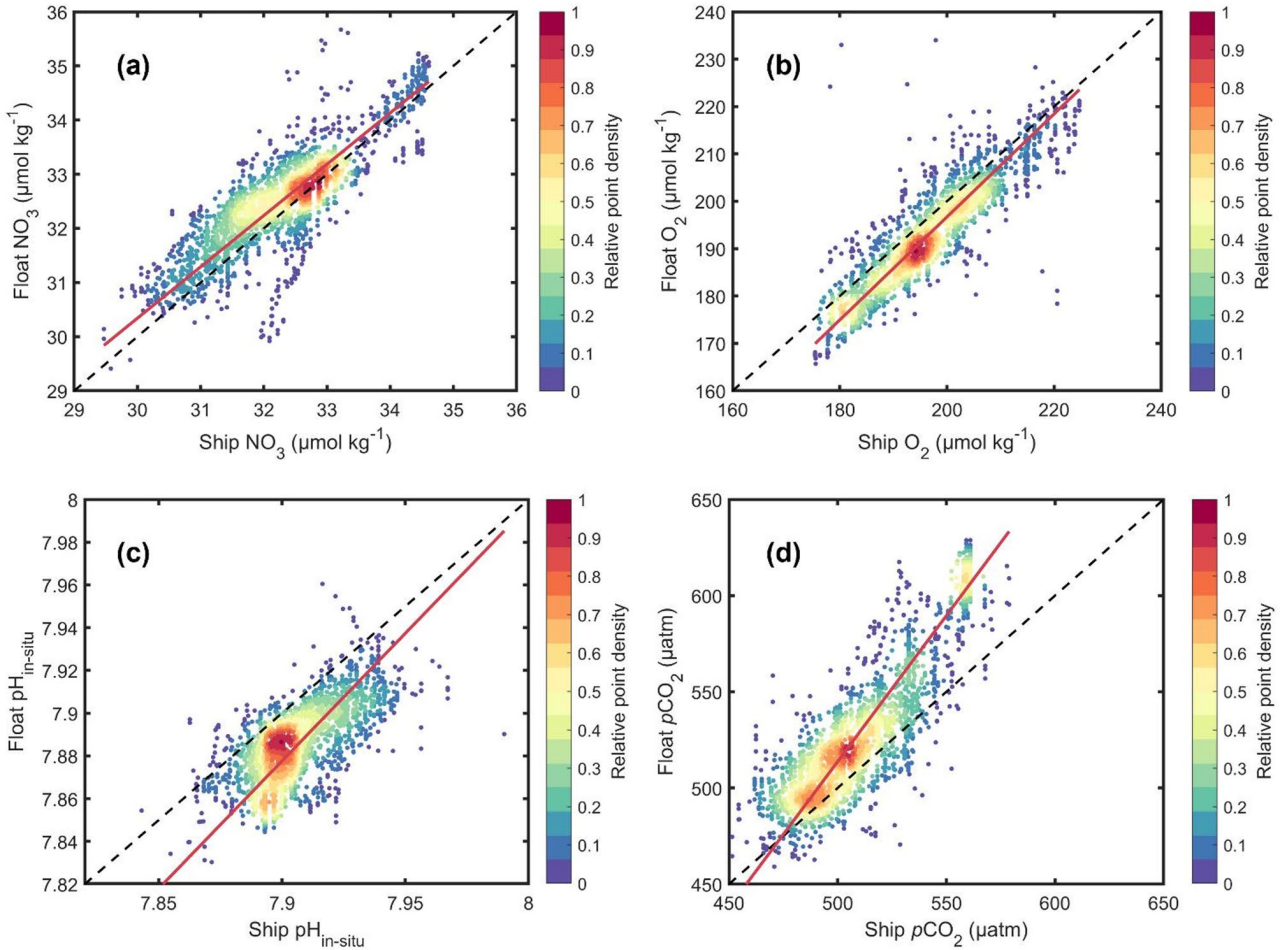
Density scatterplots of matched float-ship data in subsurface water with minimal anthropogenic carbon. Panels (a)-(d) show NO_3_, O_2_, pH_in-situ_ and *p*CO_2_. The dashed line is the 1:1 relationship (perfect agreement) and the red line is the best-fit line calculated using Deming regression. The colour of each point reflects the density of points around it.

To minimize the influence of long-term ocean acidification, we focused on deep water masses with minimal anthropogenic carbon, primarily UCDW and LCDW. These unacidified waters serve as a stable reference for assessing discrepancies between matched float-ship data. The approaches for matching float and ship data and for identifying subsurface water masses free of anthropogenic carbon are described in Methods. As a first step, density scatter plots were generated to compare NO_3_, O_2_, pH_in-situ_, *p*CO_2_ (Fig. 1) and also T, S, TA, DIC, pH_25 °C, 0 dbar_ (Supplementary Fig. 4(a), 4(b), 4(c), 4(d), 4(e)) for matched float and ship data collected in old subsurface waters (uninfluenced by anthropogenic carbon). We applied Deming regression to evaluate the consistency of the two datasets. The results show that float-based O_2_, NO_3_, T, S, and TA data agree well with the ship-based values, while pH_in-situ_, pH_25 °C, 0 dbar_, DIC and *p*CO_2_ exhibit noticeable discrepancies (Table 1).

**Table 1:** Deming regression of matched float-ship subsurface data in unacidified subsurface water for different variables.

Variable	Slope	Standard error of regression estimate^*^	Mean difference (Float-Ship)	Relative difference (%)^**^
pH_in−situ_	1.19 ± 0.06	0.0099	−0.0213	−0.27
pH_25 °C, 0 dbar_	1.48 ± 0.18	0.0075	−0.0187	−0.25
*p*CO_2_ (µatm)	1.52 ± 0.04	11.0	+ 19.8	3.86
DIC (µmol kg^− 1^)	1.37 ± 0.05	3.22	+ 5.95	0.26
TA (µmol kg^− 1^)	0.94 ± 0.03	2.32	−1.09	0.00
O_2_ (µmol kg^− 1^)	1.08 ± 0.02	3.41	−3.69	−1.89
NO_3_ (µmol kg^− 1^)	0.95 ± 0.02	0.364	+ 0.221	0.68
T (°C)	1.00 ± 0.01	0.101	0.00	0.00
S (psu)	1.08 ± 0.02	0.0112	0.00	0.00

* The standard error of regression estimate indicates the average distance between the observed values and the regression line^70^. Its unit is the same as the unit of the dependent variable. It represents a goodness-of-fit measure for regression analysis (the relationship is stronger when the value is smaller). ** Relative difference (%) is the mean percentage difference between float and ship data, calculated as (Float – Ship)/Ship × 100%.

Out of the float-based carbonate system variables, TA is estimated based on a regression algorithm^19^, pH is measured by a float sensor, and DIC and *p*CO_2_ are calculated from pH_in-situ_ and TA. Out of all the variables in Table 1, pH_in-situ_, pH_25 °C, 0 dbar_, *p*CO_2_ and DIC show the lowest consistency, with best-fit lines having slopes deviating from 1 (i.e., the slope of the 1:1 line) by more than 0.1 (i.e., slope < 0.9 or slope > 1.1). Float pH_in-situ_ is on average ~ 0.021 lower than ship pH_in-situ_. Float *p*CO_2_ is ~ 20 µatm higher than ship *p*CO_2_. In contrast, variables such as O₂, NO₃, T, and S show strong agreement between float and ship data, despite differences in sampling time and season. This consistency supports the validity of using these matched datasets for comparison. There is very good agreement for TA (Supplementary Fig. 4(c), Table 1); the mean difference in TA is −1 µmol kg^−1^ which would lead to no more than a 0.1 µatm difference in *p*CO_2_. The positive bias in derived *p*CO_2_ appears to be consistent with the negative deviation in float pH (a lower measured pH results in a higher calculated *p*CO_2_). The degree of deviation increases with decreasing pH and increasing *p*CO_2_ values.

### Discrepancies of variables at different depths between matched float-ship data in subsurface water with minimal anthropogenic carbon

**Fig. 2:**
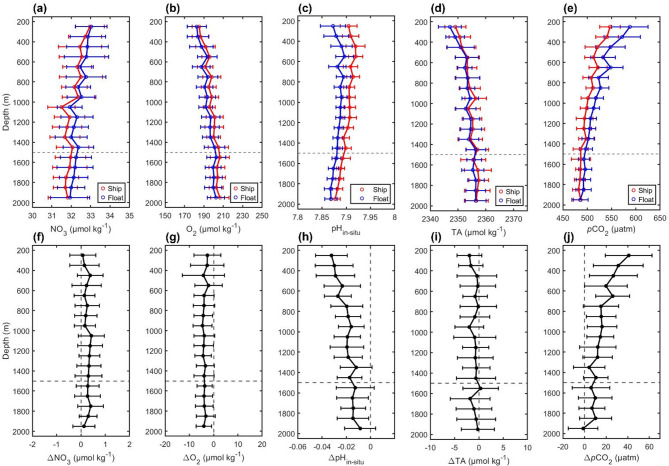
**Depth plots of NO**_3_, **O**_**2**_, **pH**_**in-situ**_, **TA and** ***p*CO**_**2**_ **from matched float-ship data in subsurface water with no anthropogenic carbon signal.** Vertical profiles of mean values: (a) NO_3_, (b) O_2_, (c) pH_in-situ_, (d) TA and (e) *p*CO_2_; and mean differences (float minus ship): (f) ΔNO_3_, (g) ΔO_2_, (h) ΔpH_in-situ_, (i) ΔTA and (j) Δ*p*CO_2_. Error bars show standard deviations of all matched float, ship or float minus ship data within each 100 m depth bin.

To further explore float pH and *p*CO_2_ deviations, we compared matched float-ship data vertical profiles for different variables. Outputs were binned in 100 m vertical intervals, to show mean values of float data and ship data against depth, as well as the mean differences between float and ship data (represented by Δ, e.g., ΔpH_in−situ_) against depth. Here we mainly compared the results of NO_3_, O_2,_, pH_in−situ_, TA and *p*CO_2_ (Fig. 2) because of the strong connection between them^71,^^72^, (for other variables, T, S, DIC and pH_25 °C, 0 dbar_, see Supplementary Fig. 5). O_2_ increases/decreases are often accompanied by *p*CO_2_ decreases/increases from photosynthesis and respiration, leading to negative correlations between Δ*p*CO_2_ and ΔO_2_.

In the depth plots, it appears that pH_in-situ_ and *p*CO_2_ have a more obvious discrepancy than other variables. Incidentally, the trend and magnitude of discrepancy in pH_25 °C, 0 dbar_ is similar to discrepancy in pH_in-situ_ (Supplementary Fig. 5). However, the variables have different units and distribution ranges (e.g., O_2_: 170–230 µmol kg^[-1^ and pH_in-situ_: 7.85–7.90), so the differences in their means cannot be directly compared. To explore the discrepancies more rigorously, we calculated the deviation degree (DD), a version of Cohen’s d, which scales the difference in the two means to the inherent variability of the data.

**Fig. 3:**
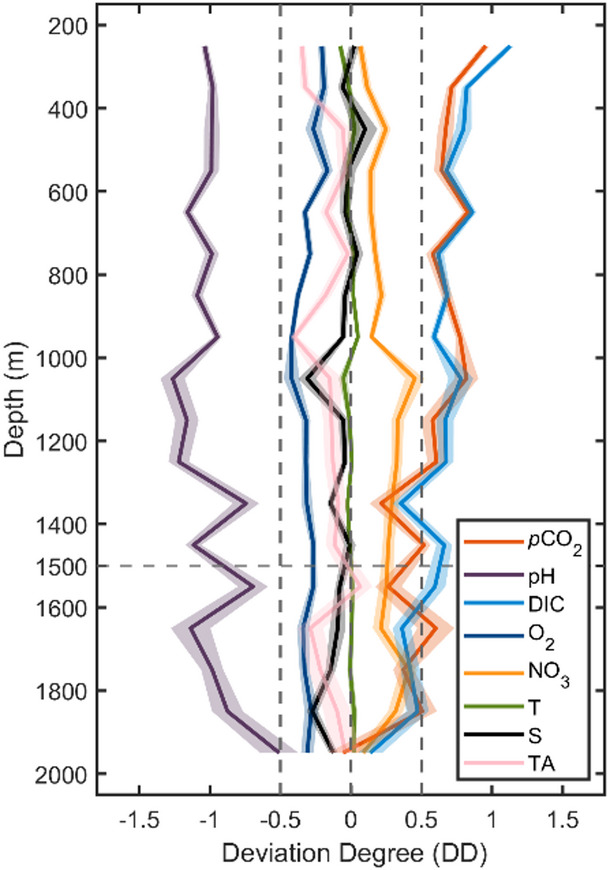
**The deviation degree (DD) of each variable (*p*CO**_**2**_, **pH**_**in-situ**_, **DIC**,** O**_**2**_, **NO**_**3**_, **T**,** S**,** TA) from matched float-ship data in subsurface water with no anthropogenic carbon in each depth interval.** The shading represents ± 1 standard error. Negative values indicate that the float mean is smaller than the ship mean and vice versa. The absolute value of DD represents the magnitude of the average difference scaled by the variability of the data.

The DDs (Fig. 3) of all variables are generally in line with our depth plots (Fig. 2): pH_in-situ_, DIC and *p*CO_2_ show the greatest degree of deviation overall. However, there are two points to note: (1) Compared with T, S and TA whose absolute DD is always less than 0.5, O_2_ and NO_3_ both show larger deviations. This suggests that the current dissolved oxygen and nitrate sensors on floats, as less well-established sensors, may not be quite as accurate as the more established temperature and salinity sensors. (2) The discrepancies in float pH_in-situ_, DIC, and *p*CO_2_ are large in the 200–1500 m depth range, with mean DD values of − 1.1 for pH_in-situ_, 0.7 for DIC, and 0.7 for *p*CO_2_. In contrast, these deviations are smaller below 1500 m, where the mean DD values are − 0.8 for pH_in-situ_, 0.4 for DIC, and 0.3 for *p*CO_2_.

### Estimated surface float ***p***CO_2_ bias

**Fig. 4:**
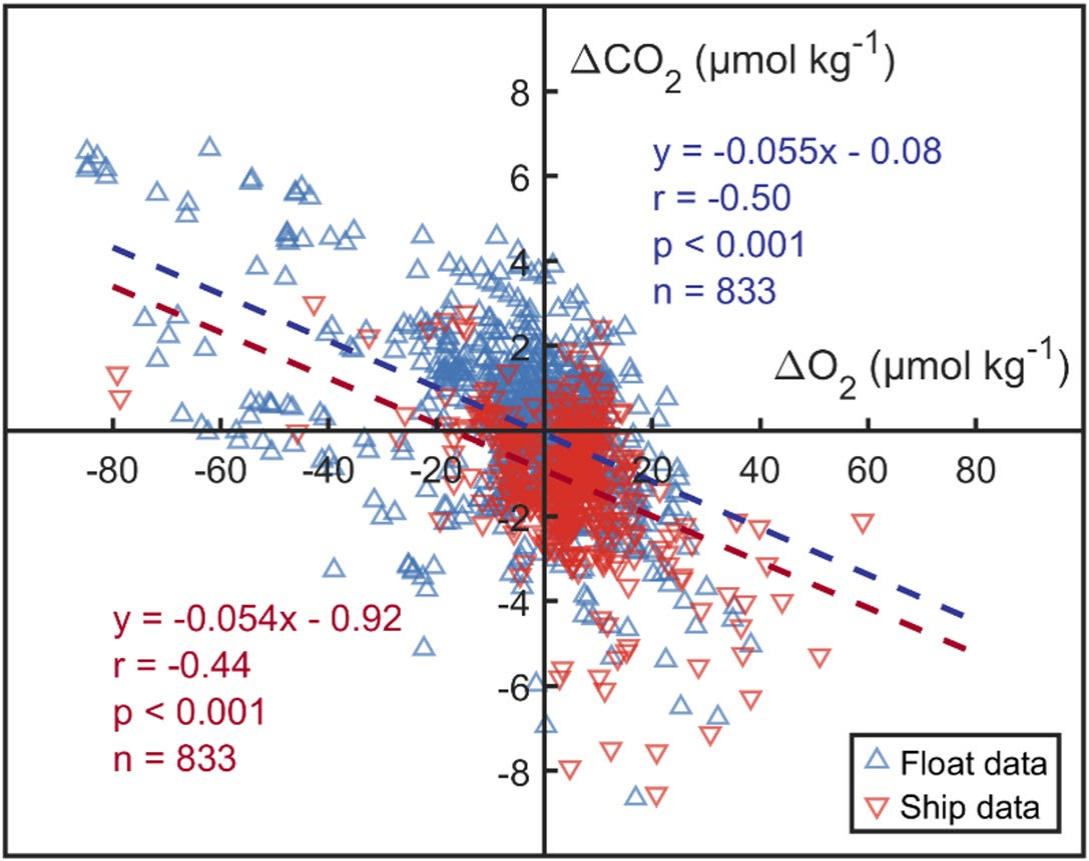
CORS plot from matched surface float data (blue) and ship data (red). The blue line is the best-fit line to float data and the red line is the best-fit line to ship data. r is the associated Pearson correlation coefficient, p denotes the significance level, and n represents the sample size. Float data (blue) is plotted first and ship data (red) second, masking the locations of many float data.

Since the calculation of CO_2_ fluxes depends on the surface *p*CO_2_, we also estimate the bias present in the surface float *p*CO_2_. We use a CORS plot (see Methods) for this. In situations where biogeochemical and physical processes like photosynthesis and upwelling strongly influence the system, the deviations of O_2_ and CO_2_ from atmospheric equilibrium demonstrate a coupled behaviour, as photosynthesis and remineralization affect carbon and oxygen in contrasting directions, according to a stoichiometric ratio. If float and ship data are both accurate then they should overlay on a CORS plot; however, while the slopes are very similar for both sets of data (−0.055 and − 0.054 respectively) the CORS plot y-intercepts are different (−0.08 and − 0.92 µmol kg^− 1^ respectively) (Fig. 4). Converting the [CO_2_] offset (0.84 µmol kg^− 1^, float minus ship) to *p*CO_2_ (in µatm), assuming average values for sea surface temperature (1℃) and salinity (35 psu), gives a difference in surface *p*CO_2_ of 14.1 µatm.

**Fig. 5:**
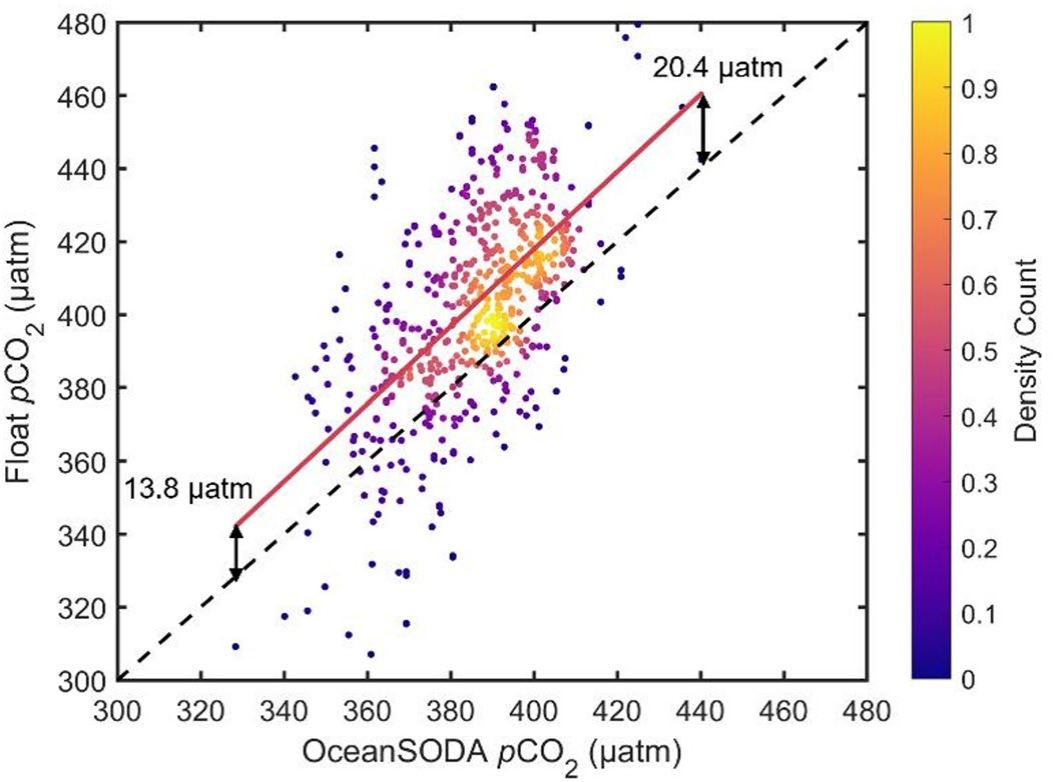
**Density scatter of matched float-OceanSODA** ***p*CO**_**2**_ **surface ocean data.** The dashed line is the 1:1 relationship and the red line is the linearly fitted line: y = 1.06 x-5.6 (*r* = 0.60). The colour of each point reflects the density of points around it.

To further investigate the float *p*CO_2_ bias in surface waters, we performed another independent comparison, between float surface data and OceanSODA output. The result is as follows: float surface *p*CO_2_ is generally higher than OceanSODA *p*CO_2_: the range of differences (between the fitting line and the 1:1 line) is 13.8–20.4 µatm, with a mean difference of 17.4 µatm (Fig. 5). This value is close to the bias of 14.1 µatm obtained from the CORS plot. Consequently, our overall estimate of the float *p*CO_2_ bias in surface waters is 14–17 µatm. This estimate is considerably larger than the current generally accepted bias of 3.6 µatm^18,^^20^. Here we would like to stress that this bias is the mean bias of 724 float profiles in the whole SO; it does not mean that every float has a deviation of equal magnitude. Comparisons with limited profiles or in localised regions are likely to give different results.

## Discussion

### Negligible influence of anthropogenic carbon and data uncertainties on detected biases

As described in the Methods section, we used a variety of methods to assess whether biases are present in float pH and therefore also in derived *p*CO_2_ and DIC. Since floats equipped with pH sensors only began to be widely deployed in 2014, their data comes from the most recent decade. The ship data, in contrast, was collected over the last 50 years (1972–2023). A potential concern is whether long-term ocean acidification-induced changes in pH and derived variables (*p*CO_2_ and DIC), stronger in the more recent data, could be misinterpreted as measurement biases, owing to differences in average year of collection of the two datasets. We took steps to address this concern and now explain why the detected biases are not a misattribution due to larger amounts, on average, of anthropogenic carbon in the float data. Any contributions from anthropogenic carbon to the stated deviations are negligible.

When estimating the surface float *p*CO_2_ bias, we utilized both CORS plots and matched float-OceanSODA datasets. In the CORS plots, ΔCO_2_ is defined as the difference between the measured *p*CO_2_ and the equilibrium (saturated) *p*CO_2_ in the surface ocean corresponding to the anthropogenic carbon at the time of measurement. While this approach minimizes the impact of long-term trends, we acknowledge that differences in air–sea equilibration timescales, particularly for upwelled waters in the Southern Ocean, may introduce some residual effects. For the matched float-OceanSODA comparisons, OceanSODA data were selected to coincide in year and month with float measurements within a spatial radius of 25 km. Consequently, these paired datasets represent synchronous observations, effectively excluding any influence from anthropogenic carbon.

**Fig. 6:**
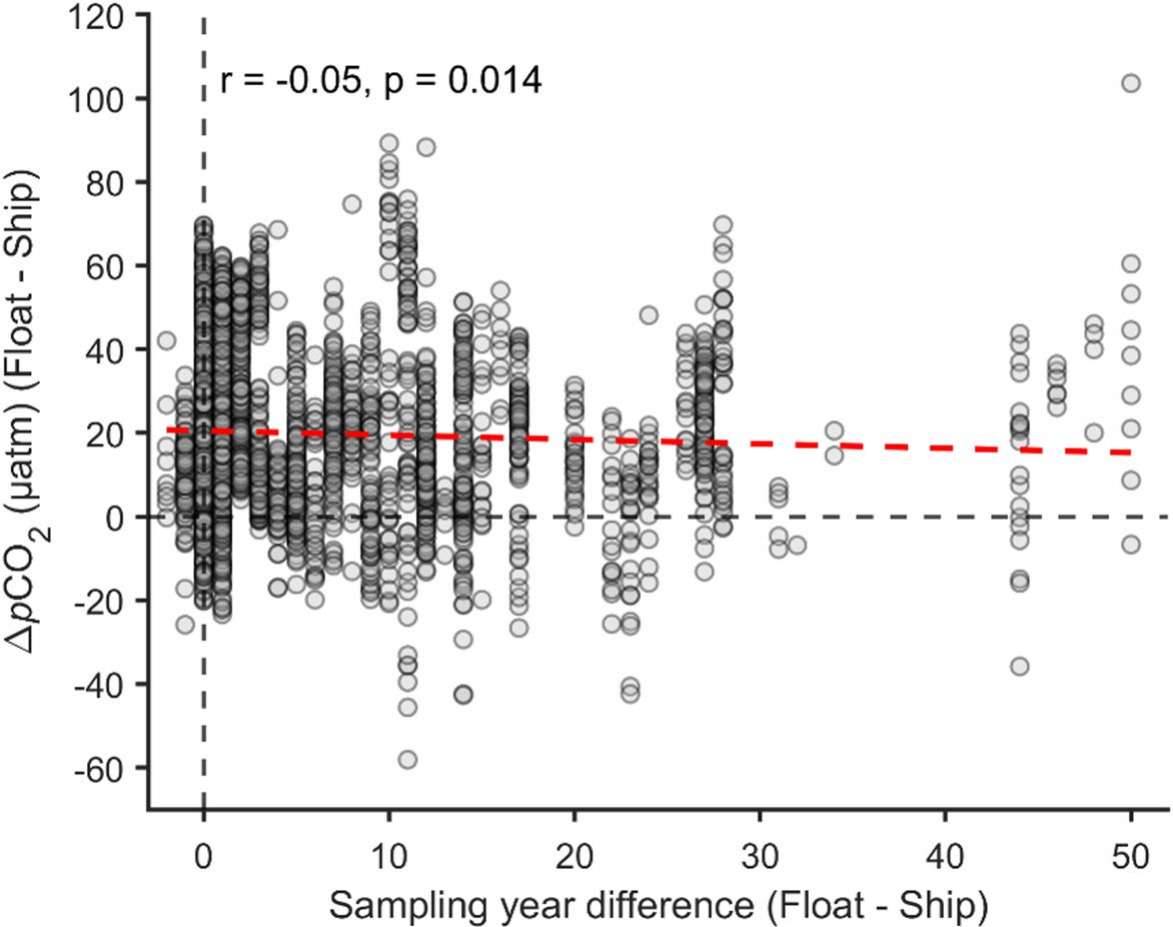
**The relationship between Δ*p*CO**_**2**_ **and sampling year difference (float minus ship) in unacidified subsurface water.** The red line is the linearly fitted line: y=−0.105x + 20.5 (*r*=−0.05, *p* = 0.014).

Another approach to address this concern was to compare ship and float data in old subsurface waters containing little or no anthropogenic carbon. We applied multiple constraints to ensure that these matched float-ship observations were obtained from water masses unaffected, or only lightly affected, by ocean acidification. Specifically, we selected only measurements taken at depths exceeding 200 m, with potential density and temperature characteristics of UCDW or LCDW, and with estimated anthropogenic carbon concentrations (based on ship data) of less than 10 µmol kg⁻¹. As a final step to ascertain that the differences identified in our selected subsurface matched dataset are not influenced by variability in sampling years, we examined the relationship between the difference in sampling years and the Δ*p*CO_2_ (float-ship). This was done for the matched float-ship data in unacidified subsurface waters (Fig. 6). The result indicates an extremely weak negative correlation (*r*=−0.05), suggesting that the float *p*CO_2_ deviations are effectively independent of differences in sampling years. Based on the outcomes of all these analyses, we categorically rule out the possibility that the float biases we present are artefacts caused by the float data being more recent than the ship data. It is also important to assess the potential role of seasonal variability. Previous studies suggest that seasonal signals in UCDW and LCDW are very small^50–52^, but we nonetheless tested this explicitly. Here, we further refined the matched float–ship data by retaining only those pairs where the float and ship observations occurred in the same season (austral summer: December–February; autumn: March–May; winter: June–August; spring: September–November) and then generated a new plot (Supplementary Fig. 6) using this reduced dataset. Compared to Fig. 2 and Supplementary Fig. 5, over 200–1500 m, the mean biases then become 17.1 µatm for Δ*p*CO_2_ (compared to 18.9 µatm when all matched pairs are used without regard to season), 4.9 µmol kg⁻¹ for ΔDIC (6.0 µmol kg⁻¹ with all matches), −0.0201 for ΔpH_in-situ_ (−0.0216 with all matches), and − 0.0178 for ΔpH_25 °C, 0 dbar_ (−0.0188 with all matches). Thus, restricting the dataset to same-season pairs changes the subsurface biases by at ~ 1.8 µatm in *p*CO_2_, ~ 1 µmol kg⁻¹ in DIC, ~ 0.0015 in pH_in-situ_ and ~ 0.001 in pH_25 °C, 0 dbar_, and the corresponding depth-resolved profiles for pH and *p*CO_2_, DIC, pH_in-situ_ and pH_25 °C, 0 dbar_ (Supplementary Fig. 6) are nearly indistinguishable from those obtained when all matched pairs are used. We therefore conclude that seasonal variability has only a minor effect on the diagnosed subsurface bias. Although our analysis focused on the mean bias, we acknowledge the broad distribution of float–ship *p*CO_2_ differences (Fig. 6). This spread reflects the combined effect of multiple factors: sensor variability, spatial–temporal heterogeneity etc. While the average bias is robust, the wide scatter suggests that not all floats behave similarly, and that environmental and methodological uncertainties play a role. A more detailed float-by-float evaluation is a suitable topic for future work.

**Fig. 7:**
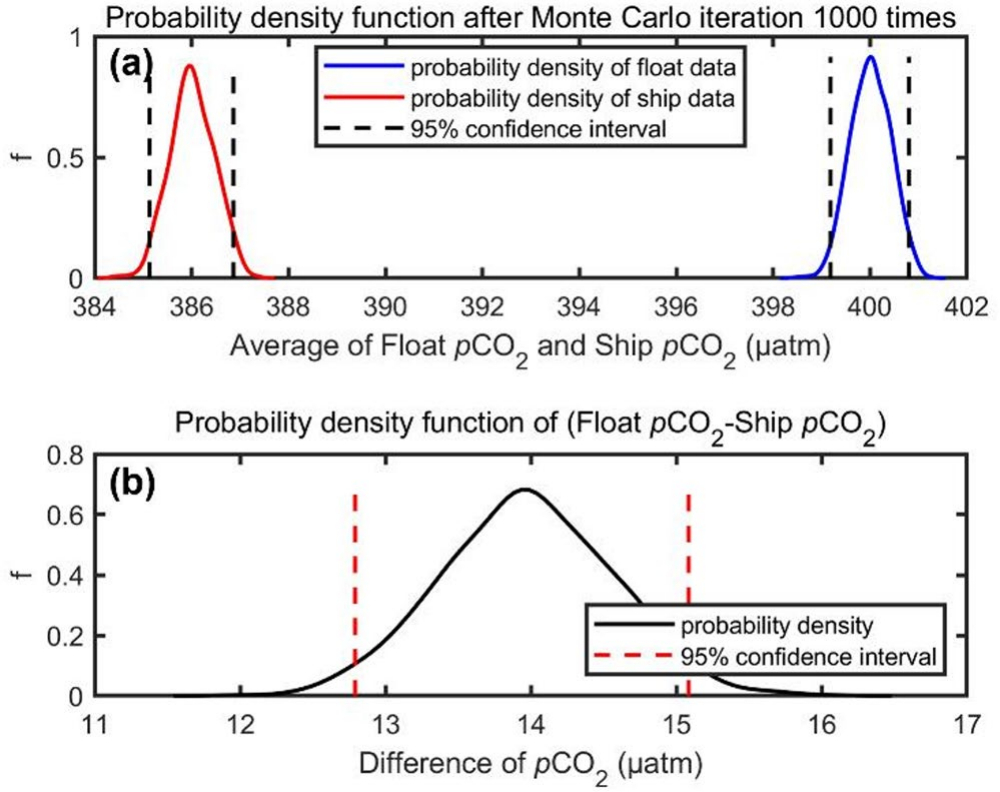
**Results of Monte Carlo calculations to test the effect of uncertainties in individual float** ***p*CO**_**2**_ **and ship** ***p*CO**_**2**_ **on the average difference between float and ship** ***p*CO**_**2**_. Assessment of (a) the impact of uncertainty in individual float *p*CO_2_ (11 µatm) and ship *p*CO_2_ (12 µatm) data on respective averages, and (b) the impact of uncertainty in individual values on the overall value of Δ*p*CO_2_ (float minus ship).

A second potential concern is whether random errors in individual measurements could be responsible for the biases seen. Data uncertainty reflects the variability or error associated with a single measurement, arising from factors such as instrument precision and random disturbances. While random errors are unavoidable, the key issue for air-sea CO_2_ fluxes is the degree of accuracy of averaged float estimates of *p*CO_2_ rather than the degree of accuracy of each individual observation^33,^^73,^^74^. We used a Monte Carlo technique (see Methods) to quantify the probable effect on overall Δ*p*CO_2_ (float minus ship) from uncertainties in individual float and ship *p*CO_2_ values. Figure 7a presents the probability density functions of average float *p*CO_2_ and ship *p*CO_2_. The effects of uncertainty in each single point of float and ship *p*CO_2_ data (11 and 12 µatm respectively) on mean Δ*p*CO_2_ are minor. At the 95% confidence level, the effect on Δ*p*CO_2_ (Fig. 7b) is less than 1.3 µatm, i.e., an order of magnitude lower than the detected bias. These results are based on the assumption that individual errors are random and independent. It does not hold for systematic biases, but that is the subject of this study. In conclusion, it is clear that, for the large datasets used in this study, the observed systematic bias cannot be attributable to the uncertainty in individual values. Based on these simulations, the confidence interval for the final estimated surface bias in float *p*CO_2_ is approximately ± 2 µatm. Accordingly, given the observed bias of 14–17 µatm and this associated confidence interval, we report the final surface bias as 15 ± 3 µatm.

### Revisiting data quality and bias of float pH and derived *p*CO_2_

pH and derived *p*CO_2_ data (hereafter collectively referred to as float carbon data) have been more frequently questioned than other variables obtained by BGC Argo floats. This is partly due to the low return rate of high-quality data^75,^^76^, from the raw measurements of the pH sensor, and partly due to the complexity of the calibration and calculation processes for pH and *p*CO_2_, some of which rely on empirical formulas without a full understanding of the underlying principles^16,^^76^. While a host of exciting discoveries have been made using float carbon data^73^, some concerns remain about its overall reliability.

Our results provide evidence that float carbon data exhibits considerable biases, not only within the surface layer but also throughout the entire vertical profile. The surface *p*CO_2_ bias found by this research 15 ± 3 µatm is significantly larger than the previously recognized bias (3.6 µatm) suggested by Gray et al.^18^, but close to a regional comparison result (14 µatm)^27^. It is also much larger than biases of 0.003 in float pH and 3.2 µatm in float *p*CO_2_ attributed to a lag in the response of the float O_2_ sensors^77^ – our analyses suggest that correction of this lag, while helpful, would only remove a small fraction of the total biases we detect in float pH and *p*CO_2_. The observed subsurface *p*CO_2_ bias (mean Δ*p*CO_2_ of 18 µatm between 200 and 1500 m depth, Fig. 2) is even more pronounced than the surface bias. The pH and *p*CO_2_ biases we have found in the subsurface (200–1500 m depth) imply that there may be issues with the current calibration process (described in detail in the Methods section) for float pH data. Using the mismatch between float and algorithm pH at 1500 m depth to adjust the whole profile pH data may therefore be insufficient or not always appropriate. The pH and *p*CO_2_ consistency in the 1500–2000 m depth range indicates that the correction procedure works well below 1500 m depth. However, the biases identified in the 200–1500 m depth range may occur because the difference between float measured pH and actual pH over the whole profile is not always the same as at 1500 m depth. Our recommendation is to add a series of cross-reference points above 1500 m depth for the float pH correction, and to use the differences at multiple depths to adjust pH, rather than only the difference at 1500 m, which aligns with the conclusion presented by Wimart-Rousseau^30^. Further examination and improvement of the float pH adjustment procedure is likely to be necessary in order to obtain more accurate pH and derived *p*CO_2_ values.

### Implications for flux calculations

If the surface *p*CO_2_ bias found by this research 15 ± 3 µatm is considered in the calculation of SO carbon fluxes based on float *p*CO_2_ data, the recalculated SO net annual carbon uptake will be much higher. In this context, negative flux values represent CO₂ uptake by the ocean (ingassing), and positive values represent CO₂ release to the atmosphere (outgassing). Recalculation of the SO fluxes is beyond the scope of this study, but we give an estimate here, based on the results from Bushinsky et al.^20,22^. They found that a 4 µatm reduction in the SOCCOM product led to an increase of −0.21 Pg C yr⁻¹ in the estimated Southern Ocean carbon sink^20^. Assuming that the effect of bias on flux is proportional, our conclusion of a bias of 15 ± 3 µatm implies a reduction of the float-based flux from + 0.35 Pg C yr^− 1^ to between − 0.28 Pg C yr^− 1^ and − 0.60 Pg C yr^− 1^, closer to flux estimates based on ship data (−0.38 Pg C yr^− 1^) and aircraft data (−0.53 Pg C yr^− 1^)^22–24^. This rough estimate suggests that the bias reported in this study could explain a large fraction of the current large discrepancy between fluxes based on float data versus fluxes based on other observations. This suggests that previous studies using float data have underestimated carbon uptake by the SO due to the biases in float pH and derived *p*CO_2_.

Despite the current biases in the BGC Argo float pH and derived *p*CO_2_ data, the value of the approach is without question. Float data expands the area and time coverage of data^3,^^78^. The significant advances in observational capabilities with float data have contributed to an expanded understanding of processes acting at smaller scales^9^. Incorporating the insights from our research into the design of a future calibration process for float pH will further improve the quality of the pH data. At the same time, our results emphasize the value of continuing to collect high quality ship-based measurements as a reference for the larger volumes of autonomously collected data. Although various autonomous observation platforms have been developed in recent years, their data is less certain and will continue to need to be calibrated against ship observations. A combination of high-accuracy ship data and improved high-volume, wide-coverage float data will provide a deeper understanding of carbon dioxide uptake/release in the SO and a better quantification of the role of SO in the global carbon cycle.

### Limitations and future directions

Our findings are based on data analysis and are subject to several inherent limitations. In the following, we describe some of these limitations and suggest directions for future research that could complement and build upon the work described here.

A first limitation is that our analysis is carried out within the inorganic carbonate system which can be affected by internal thermodynamic inconsistency^79^. The subsurface comparisons rely on GLODAP as a reference dataset, within which pH is most often calculated from measured DIC and TA instead of being measured directly using spectrophotometric techniques^79^. Previous studies have revealed a pH-dependent offset between DIC&TA-derived pH and spectrophotometrically measured pH^17,29,80,81^. This suggests a scenario in which the entire offset found in our study arises not from bias in float pH but instead from biases in matched GLODAP pH, because the latter are computed incorrectly from measured DIC and TA.

However, two independent lines of evidence argue against this being the main reason for the biases we find. First, float-derived DIC is systematically higher than ship DIC according to the subsurface comparison (Fig. 3 and supplementary Figs. 4 d and 5 d) while float TA agrees closely with ship TA. The study by García-Ibáñez et al. has shown that when high-quality spectrophotometric pH and accurate TA are used, DIC calculated from pH and TA is internally consistent with measured DIC and exhibits little systematic bias^82^. It would seem therefore that the sizeable DIC offsets we find cannot be explained solely by errors in GLODAP pH, because if that were the case then float and ship DIC should match. Second, our estimate of the surface *p*CO_2_ bias relies not only on comparison to GLODAP but also on comparison to the OceanSODA data set, where the latter is constrained by direct surface CO₂ measurements in the SOCAT database^23^ (i.e. does not involve any calculations of pH from DIC and TA). We obtain similar high biases (both ~ 15 µatm) from the comparisons to GLODAP and OceanSODA. This indicates that the observed discrepancies are not solely due to carbonate system thermodynamic inconsistency. Future work could include comparisons of float pH to matched spectrophotometrically measured ship pH sometime after float deployment, for instance by determining which cruises in the GLODAP dataset include direct measurements of pH and then looking for crossovers with float tracks.

A second limitation concerns the way surface and subsurface biases are linked in this study. Because the spatial–temporal coverage of co-located ship measurements is too limited to apply the same float–ship matching strategy at the surface and at depth, we diagnosed the bias from a subsurface float–ship comparison in old water with minimal anthropogenic carbon signal and then use an independent surface analysis based on CORS and OceanSODA to quantify how this bias manifests at the surface. Considering that the standard SOCCOM processing applies a single offset, estimated near 1500 m, to the entire pH profile, it is reasonable to expect surface and subsurface pH biases to be related rather than independent; our results are consistent with this picture, but variable concentrations of anthropogenic CO_2_ and seasonality in surface waters make it harder to analyse biases there. Additional co-located ship–float observations with full-depth carbonate-system measurements (including ship-measured pH) would provide the most direct test of the bias patterns inferred here.

A third limitation is that, although we have explicitly examined and discussed a wide range of potential contributors to the observed discrepancies, it is impossible to prove an absence and so we cannot definitively exclude other unanticipated contributors to the diagnosed biases. Our analyses considered: (i) anthropogenic carbon, (ii) measurement uncertainties in both ship and float data, (iii) the accuracy of algorithm-estimated float TA, and (iv) effects of interannual variability as well as seasonality. None of these factors were found able to explain the magnitude and vertical structure of the pH, DIC and *p*CO_2_ offsets. However, the inorganic carbon system and the float *p*CO_2_ processing are complex, and with the currently available data we cannot separate sensor-related effects (e.g. drift, ageing, pressure response) from limitations of the adjustment procedure or from other as yet unidentified sources of bias. Clarifying the underlying mechanisms will require targeted laboratory and field experiments on the float pH sensor. Further development and intercomparison of alternative float pH adjustment schemes is also recommended.

## Conclusions

This study investigated the accuracy in the SO of pH and derived *p*CO_2_ data from BGC Argo floats by comparing it to high-quality ship data from the GLODAP dataset. A first comparison, restricted to old subsurface waters in order to exclude effects of anthropogenic carbon, identified systematic differences in float pH and derived *p*CO_2_. On average, float pH was ~ 0.021 units lower than ship-based values, *p*CO_2_ was ~ 20 µatm higher, whereas other variables (T, S, NO_3_, O_2_) showed good agreement. A second comparison used a CORS plot to investigate float *p*CO_2_ bias in surface waters, yielding a result of 14 µatm. A similar offset (range: 14–20 µatm, mean 17 µatm) was obtained by an independent method, comparing float surface *p*CO_2_ to OceanSODA results for the same location, month and year. Float *p*CO_2_ values are derived from both float pH and algorithm-estimated TA, but float-ship TA comparison showed that the influence of TA bias on discrepancies can only be very small (order 0.1 µatm). We consider a previously unrecognized bias in float pH to be the most plausible explanation for the observed *p*CO_2_ offsets. This bias is larger above 1500 m depth and smaller below 1500 m depth, which suggests that the current float pH correction process (adjusting the entire float pH profile by comparing float-measured to algorithm-estimated pH at 1500 m depth) may not fully account for some depth-dependent offsets. Improving the existing float pH correction method could lead to more accurate pH and *p*CO_2_ data, and thus more robust quantification of carbon uptake in the SO. Further work is necessary to clarify the mechanisms underlying this pH bias. Overall we estimate that float surface *p*CO_2_ is biased high by 15 ± 3 µatm. Simple scaling arguments based on published flux sensitivities suggest that reducing float surface *p*CO_2_ downward by this amount would largely account for the currently debated large discrepancy between float-based SO carbon flux estimates and those derived from other platforms.

## Supplementary Information

Below is the link to the electronic supplementary material.

Supplementary Material 1

## Data Availability

The ship (GLODAP) data were obtained from https://www.glodap.info (last access: 05 February 2024). The float data were downloaded from https://library.ucsd.edu/dc/object/bb51655605, 2023-12-20 snapshot. The OceanSODA data were downloaded from https://www.ncei.noaa.gov/data/oceans/ncei/ocads/data/0220059/.

## Code availability

Matlab code for this study is available upon request to C.Z.
