# Supplementary material for: A systematic bias in float pH leads to overestimation of derived pCO2 and underestimation of carbon uptake by the Southern Ocean
Source: Sci Rep. 2026 Mar 17;16:13929. doi: 10.1038/s41598-026-43863-4 (PMC13133385; doi:10.1038/s41598-026-43863-4)
Supplement: Supplementary file 1 — Supplementary Material 1 [file 41598_2026_43863_MOESM1_ESM.docx]

**Supplementary Information:** **A systematic bias in float pH leads to overestimation of derived *p*CO_2_ and underestimation of carbon uptake by the Southern Ocean**

Chuqing Zhang^1*^, Yingxu Wu^2^, Peter J. Brown^3^, David Stappard^1^, Amavi N. Silva^1,4^, and Toby Tyrrell^1^

^1^University of Southampton, National Oceanography Centre Southampton, Southampton, UK

^2^Polar and Marine Research Institute, Jimei University, Xiamen, China

^3^National Oceanography Centre, Southampton, UK

4GEOMAR Helmholtz Centre for Ocean Research Kiel, Kiel, Germany

*Correspondence to*: Chuqing Zhang ([Chuqing.Zhang@soton.ac.uk](mailto:Chuqing.Zhang@soton.ac.uk))


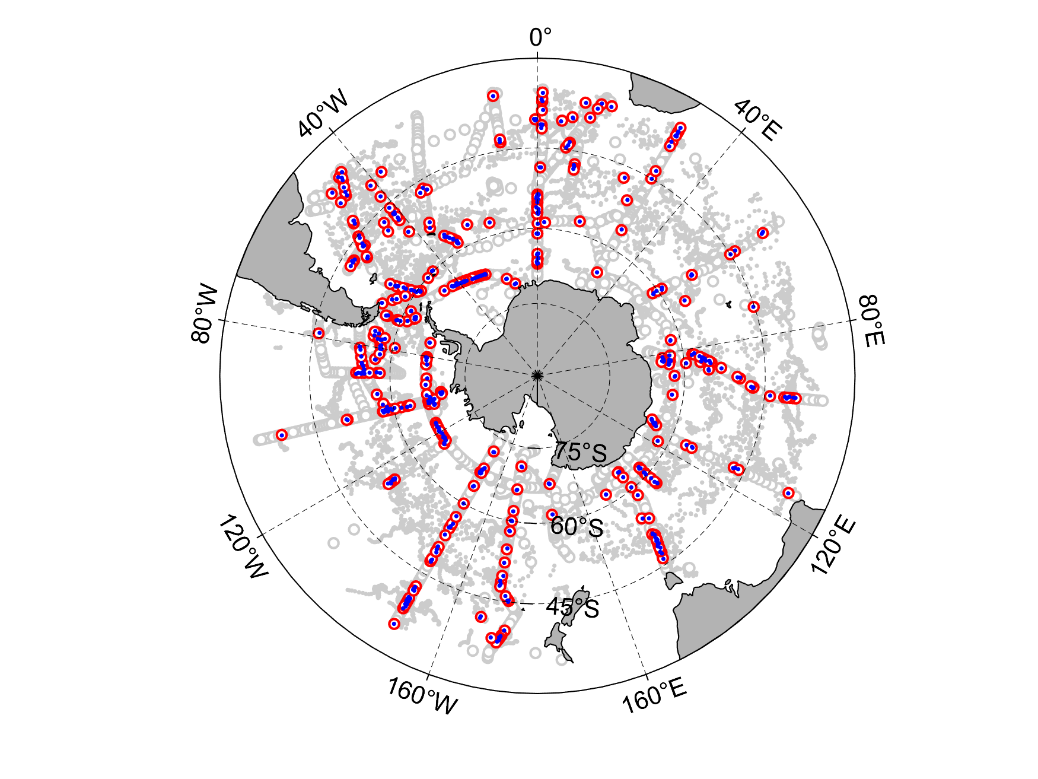


**Supplementary Figure 1. Locations of matched float (blue dots) and ship profiles (red circle) (maximum matching distance of 25km).** Grey dots and circle show locations of unmatched float profiles and ship profiles.


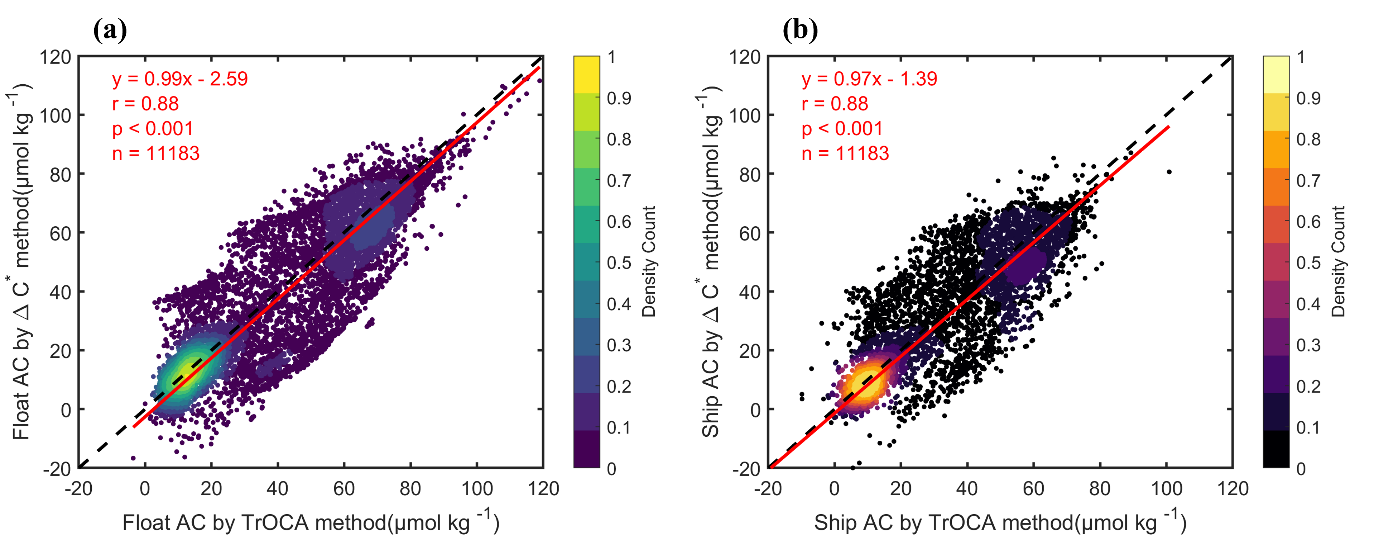


**Supplementary Figure 2. Density scatterplots of anthropogenic carbon calculated by TrOCA method and ∆C* method of matched (a) float data, (b) ship data.** r is the associated Pearson correlation coefficient, p denotes the significance level, and n represents the sample size. The dashed line is the 1:1 relationship and the red line is the result of Deming regression. The colour of each point reflects the density of points around it.


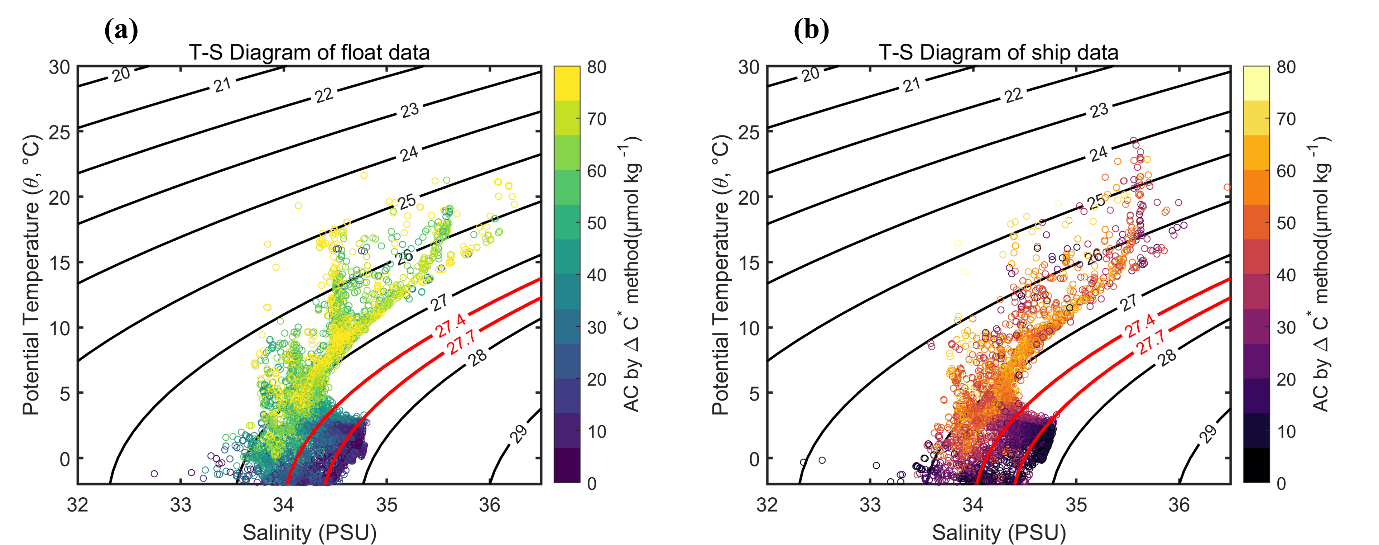


**Supplementary Figure 3. T–S diagrams overlaid with anthropogenic carbon concentrations estimated by the ∆C* method for (a) float data and (b) ship data.** The background contours represent lines of equal potential density. Red lines highlight the 27.4 and 27.7 contours, which are used to delineate unacidified water masses. The colour of each point indicates the estimated anthropogenic carbon concentration (μmol kg⁻¹).


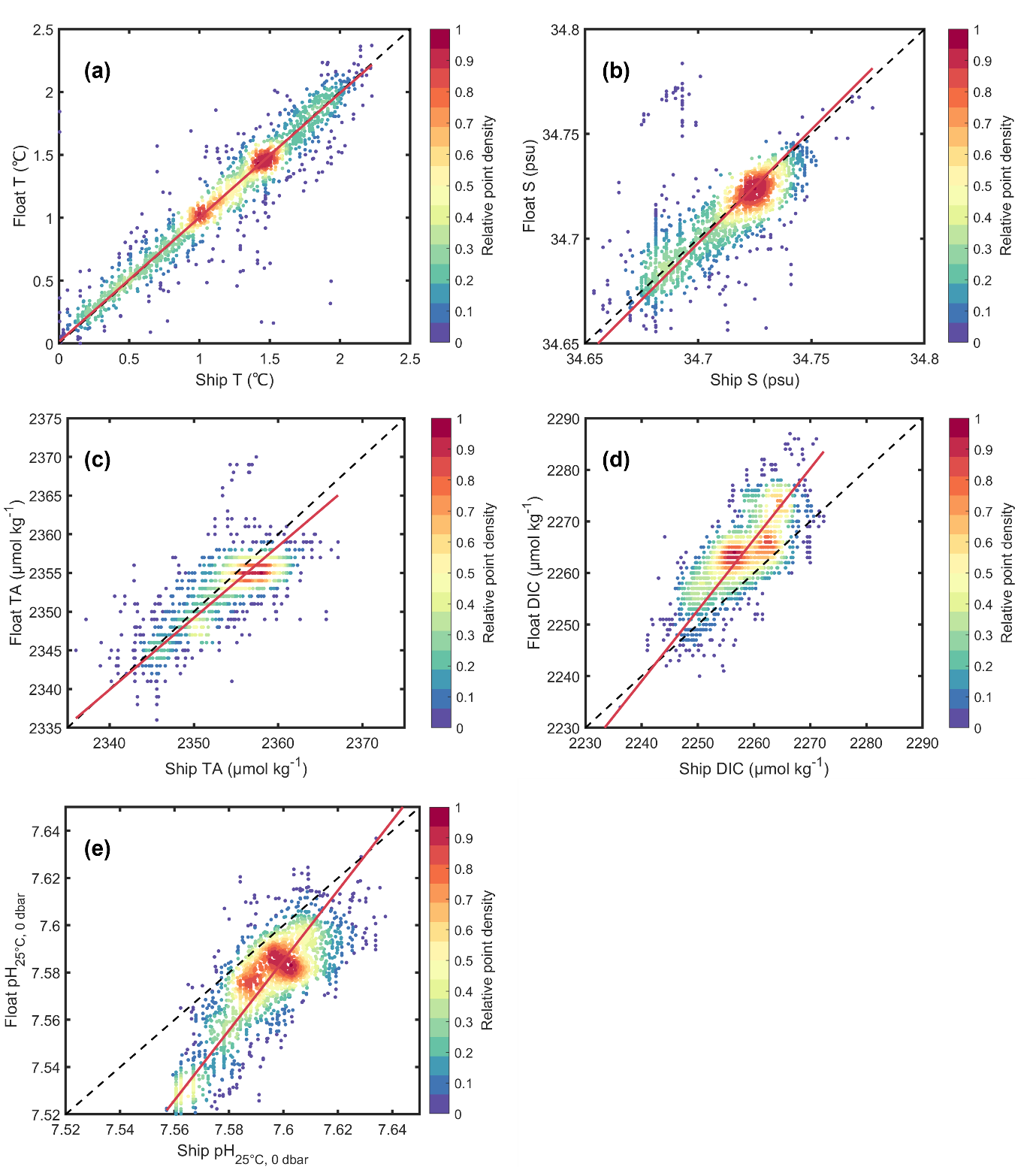


**Supplementary Figure 4.** **Density scatterplots of matched float-ship data in unacidified subsurface water.** Panels (a)-(e) show T, S, TA, DIC and pH_25°C, 0 dbar_. The dashed line is the 1:1 relationship and the red line is the result of Deming regression. The colour of each point reflects the density of points around it.


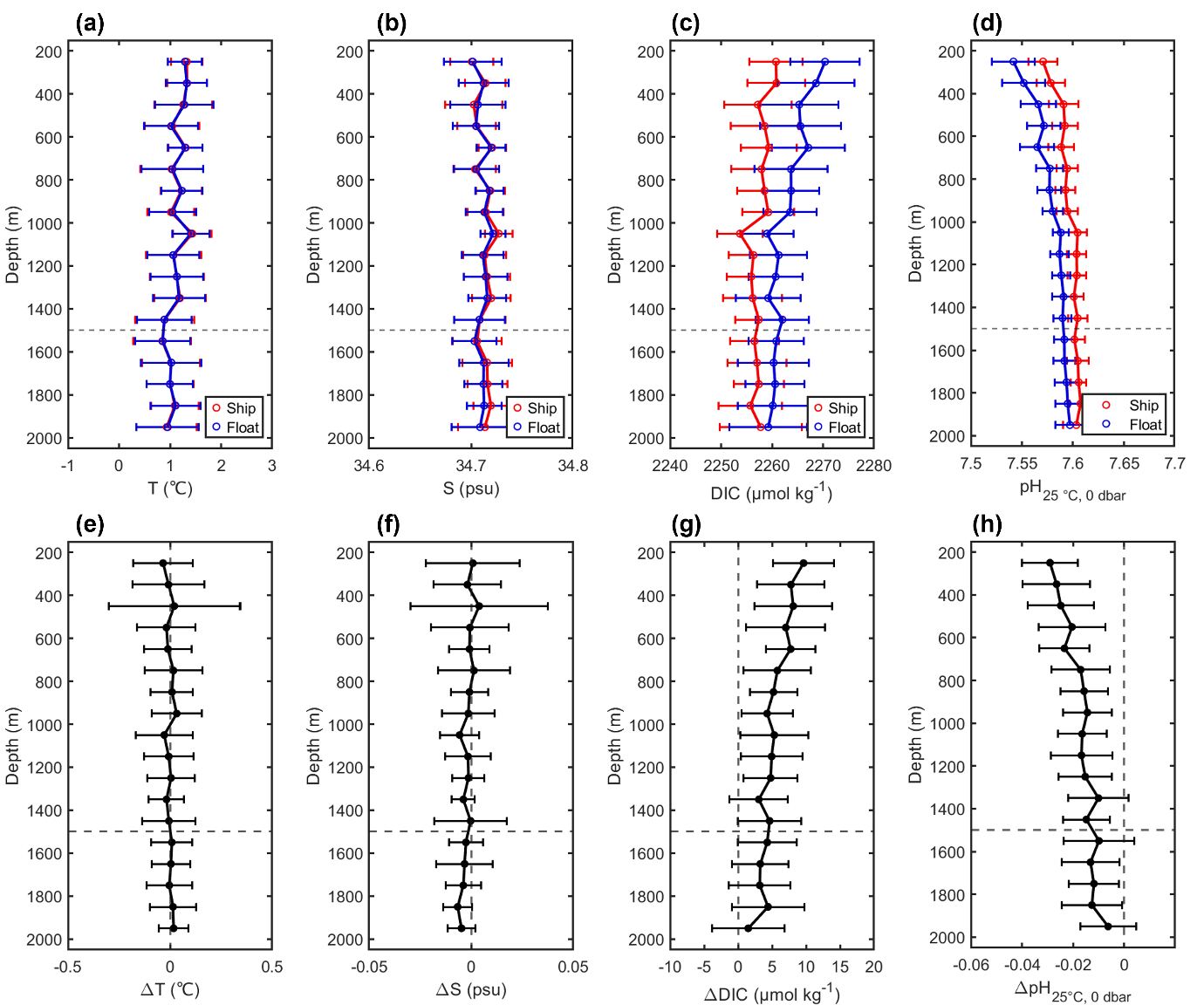


**Supplementary Figure 5. Depth plots of T, S, DIC and** **pH_25°C, 0 dbar_ from matched float-ship data in unacidified subsurface water.** Vertical profiles of mean values (a, b, c and d; T, S, DIC and pH_25°C, 0 dbar_) and mean differences (e, f, g and h; ΔT, ΔS, ΔDIC and ΔpH_25°C, 0 dbar_). Error bars show standard deviations of all matched float, ship or float minus ship data within each 100 m depth bin.


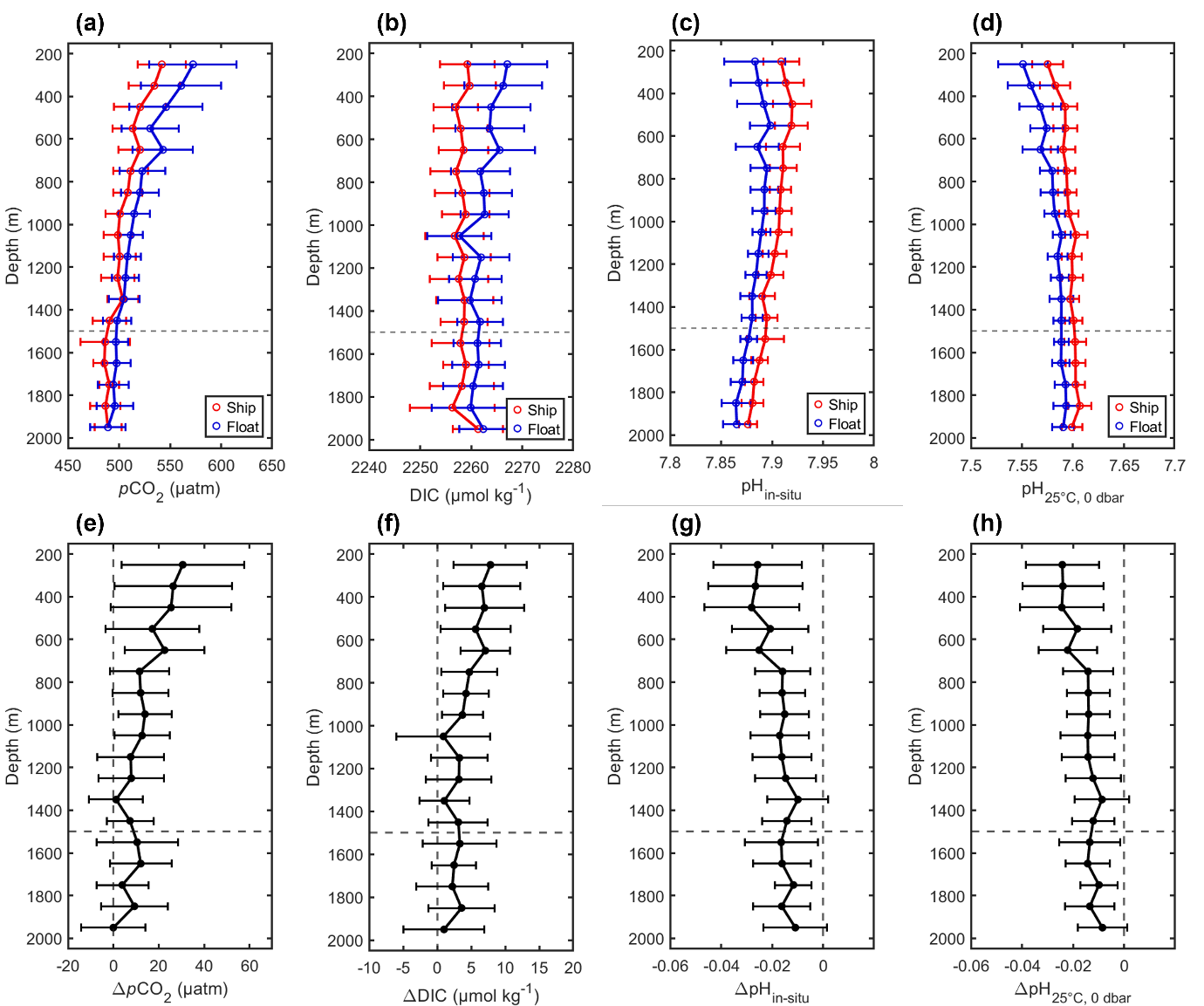
 **Supplementary Figure 6. Depth plots of *p*CO_2_, DIC, pH_in-situ_ and pH_25°C, 0 dbar_ from matched float-ship data in unacidified subsurface water, restricted to same-season pairings.** Vertical profiles of mean values (a, b, c and d; *p*CO_2_, DIC, pH_in-situ_ and pH_25°C, 0 dbar_) and mean differences (e, f, g and h; Δ*p*CO_2_, ΔDIC, ΔpH_in-situ_ and ΔpH_25°C, 0 dbar_). Error bars show standard deviations of all matched float, ship or float minus ship data within each 100 m depth bin.
